# Analysis of the impact of public health emergencies on the dry bulk shipping market

**DOI:** 10.1371/journal.pone.0265216

**Published:** 2022-04-06

**Authors:** Jing Liang, Tianlun Dai, Shuai Sun

**Affiliations:** Department of College of Transportation Engineering, Dalian Maritime University, Dalian, China; University of South-Eastern Norway USN School of Business: Universitetet i Sorost-Norge USN Handelshoyskolen, NORWAY

## Abstract

A structural vector autoregressive model and spillover index analysis based on generalized prediction error variance decomposition were used to explore the impact of public health emergencies on the dry bulk shipping market and provide suggestions for addressing the impact of public health emergencies. Moreover, the risk fluctuation and spillover of the dry bulk shipping market during public health emergencies were analyzed to understand the ways in which public health emergencies impact the dry bulk shipping market and to quantify the impact intensity. In related studies, the influence of the international crude oil price index and dry bulk ship port berthing volume were also considered. The results show that considering the immediate impact, the increase of newly confirmed cases of COVID-19 has a significant impact on the dry bulk shipping market, which lasts for more than 3 weeks and is always a negative shock. Different types of public health emergencies have different effects on the dry bulk shipping segmented shipping market. Dry bulk shipping companies should fully understand the development of public health emergencies, make full use of risk aversion forecasting tools in financial markets and make deployments for different situations.

## 1 Introduction

### 1.1 Research background and purpose

The first public case was reported on December 31, 2019, and the World Health Organization declared the 2019 novel coronavirus (COVID-19) a global pandemic on March 11, 2020. Although the severity of the pandemic in different countries and regions is different in terms of the number of confirmed cases, each country and region has been negatively affected, and the negative impacts continue to spread.

A safe and stable external environment is the foundation of the sound development of each industry’s economy. The prevention of a series of abrupt public health events has always been an important issue for every economy and industry. In recent years, with the global outbreak of major public health emergencies such as the SARS pandemic, the Zika virus epidemic, and the COVID-19 pandemic, public health emergencies have received increasing attention because of their high degree of suddenness, low regularity and high transmission and their strong impact on economic development. As an important bridge of economic globalization, dry bulk shipping actively helps construct the “Sea route of the new Silk Road”. The dry bulk shipping market, which has always been closely related to the development of economic globalization, is sensitive to the impact of sudden public health events that impact the global economy.

In this context, it is conducive to the sound development of dry bulk shipping, the globalization of service economy in the perspective of goods, capital, technology, personnel circulation and provide power and space for economic growth by analyzing the impact of public health emergencies on the dry bulk shipping market and the risk volatility of the dry bulk shipping segment market.

### 1.2 Literature review

#### i) Impact of public health events on markets in general

According to current research and the impact of public health emergencies on markets in general, Baldwin and Tomiura predicted that the outbreak of COVID-19 would have an impact on both supply and demand of the global economy and a significant impact on international trade [1]. Açikgöz Ö and other studies posit that COVID-19 has severe adverse effects on employees, customers, supply chains and financial markets and will most likely cause a global economic recession [2]. Feyisa H L’ believes that the COVID-19 pandemic is an increasing economic threat [3]. Maital S and others believe that under COVID-19, the resilience of our economic, social and medical systems will be severely tested [4]. Craven M and others believe that the COVID-19 public health emergency has had a growing impact on the global economy [5]. Vidya C T and others believe that under the influence of COVID-19, there has been a drastic decline in trade of most of the economies [6]. McKibbin W and others predicted that even a contained outbreak could significantly impact the global economy in the short run [7]. Weiss M and others believe that the pandemic is having a noticeable impact on global economic growth. Estimates thus far indicate that the virus could trim global economic growth by as much as 2.0% per month [8].

In general, the COVID-19 public health emergency has had a huge impact on the smooth operation of the global economy. The above documents provide many ideas for this article to explore the path analysis of the impact of public health emergencies on the dry bulk shipping market.

#### ii) Impact of public health events in shipping markets

According to current research and the impact of public health emergencies on shipping markets, Zhang Yongfeng and others believe that the daily increase of the COVID-19 pandemic has a significant Granger causality with the BDI index [9]. Hakan Yilmazkuday believes that the increase of confirmed cases of COVID-19 will reduce BDI by 7% in 7 days [10]. Xu Peihong and other researchers believe that the impact of the pandemic on Yangtze River shipping is generally controllable and short-term, mainly in the first quarter [11]. Wan Chengpeng and other researchers believe that the negative impact of the COVID-19 pandemic will superimpose the impact of the Spring Festival off-season, which has a great impact on the international shipping industry [12]. Ge Yingen and others found that the impact of the COVID-19 pandemic on the shipping industry is reflected in its impact on market supply and demand [13]. Liu Zhibiao and other researchers believe that the outbreak of COVID-19 will cause a rupture in China’s import and export industry supply chain in the short term [14]. Liu Fang and other researchers believe that the COVID-19 pandemic has a huge impact on transportation production, construction and other fields [15]. Menhat and other researchers believe that the COVID-19 pandemic will impact shipping by affecting blockade decisions between countries (regions) [16]. Shailender and other researchers believe that the COVID-19 pandemic has had a shock wave effect on global shipping [17]. Notteboom and others believe that the external impact of the pandemic is mainly reflected in how the shipping industry adapts to the complex supply chain and how ports respond to cargo handling [18]. Zhou Jian believes that the impact of the COVID-19 pandemic on the transportation industry is short-term and will not cause long-term trend changes [19]. Xu L and other researchers have investigated the gaps that occur in the shipping trade between China and different regions, and they believe that government prevention and control measures regarding the pandemic have a negative impact on export trade, while import trade increases accordingly [20]. Notteboom T E and other studies believe that the COVID-19 pandemic is having a major impact on the economic activity in seaports [21]. March D and other researchers believe that the ocean ship-based activities have been impacted due to severe restrictions on human movements and changes in consumption [22]. Michail N A and other studies believe that COVID-19 is directly affecting the dry bulk and the dirty tanker segments [23].

The impact of public health emergencies on the shipping market is sudden and severe. The impact of different public health emergencies on the risk volatility and spillover of the dry bulk shipping market is less researched.

### 1.3 Research path introduction

**Fig 1:**
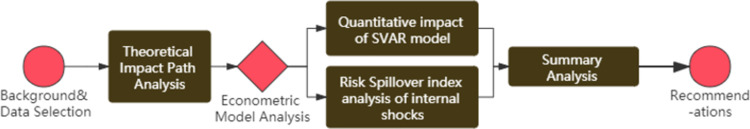
Structure diagram of the article’s analysis.

As shown in Fig 1, this paper will analyze the impact of the public health emergency of the 2019 COVID-19 pandemic on the dry bulk shipping market and provide relevant suggestions for dry bulk shipping enterprises to deal with impacts and crises. Based on this, the first outbreak of COVID-19 in dry bulk trade demand countries in 2019 and the first outbreak of Zika in dry bulk trade supply countries in 2016 were studied. The risk spillover level and the internal risk transfer of external risk in each market segment of the three-subdivision ship market in the dry bulk shipping market were investigated when the two public health emergencies occurred.

## 2 Data sources and analysis

The COVID-19 pandemic was selected to explore the effect of the public health emergency on the dry bulk shipping market.

### 2.1 Data selection for investigating the impact of the COVID-19 pandemic

The Baltic Dry Bulk Freight Index (BDI index) was selected to reflect the overall development of the dry bulk shipping market. Newly diagnosed pneumonia cases (COVID-19) were selected to reflect the development of the COVID-19 pandemic. The impact of the COVID-19 pandemic on the dry bulk shipping market was explored through the interaction between the two. At the same time, to perfect the exploration of the impact angle, the supply-side European Brent crude price index (EBSP) reflects the influence of the supply-side international crude oil market and the demand-side Clarkson Company’s statistics of the daily port calls of bulk carriers above 65,000 dwt (Dead Weight Tonnage) reflect the actual demand of the dry bulk shipping market to further analyze the impact of the COVID-19 pandemic on the international dry bulk shipping market. The number of port calls per day reflects the real state of the dry bulk shipping market to a certain extent.

The COVID-19 pandemic in China gradually faded away after the “unblocking” of Wuhan. The major trading powers adopted trade intervention with a relatively lifted ban. The dry bulk shipping market and related enterprises have gradually resumed under the series of shocks [24]. Furthermore, the COVID-19 pandemic is not reflected very accurately by the crude oil market as the upstream market of the dry bulk shipping market and the strong fluctuation of the relevant market. Considering the above factors, combined with the analysis of measurement software EViews, the measurement time span from January 3, 2020 to April 8, 2020 is selected.

### 2.2 Data selection for investigating the risk transfer in the dry bulk shipping market segment

The BCI, BHI and BPI indices issued by the Baltic shipping Union represent the Capsize dry bulk market, the (Super) Handysize dry bulk market and the Panamax dry bulk market, respectively. The outbreak of the COVID-19 pandemic and the study period of the Zika virus were selected from January 3, 2020 to April 8, 2020 and February 1, 2016 to July 31, 2016, respectively. In February 2016, the World Health Organization (WHO) announced that the Zika epidemic in Brazil was a public health emergency of international concern, so February 1, 2016, was chosen as the starting time of the impact period of the Zika epidemic in Brazil. In September of the same year, the WHO announced that no laboratory-confirmed Zika virus cases were found in Brazil during the Olympic Games (August and September 2016). In November, it announced that the pheic status of the Zika virus epidemic in Brazil was lifted [25]. To make the time periods of the two exploration periods as close as possible, July 31, 2016, was selected as the end of the impact period of the Zika epidemic in Brazil. The reasons for choosing the COVID-19 pandemic area are listed above.

## 3 impact path analysis

### 3.1 Dry bulk shipping and interindustry transmission fluctuations from the perspective of policy restrictions

Public health emergencies are closely related to population mobility and directly affect public consumption, business tourism transportation and logistics and other industries. With population flows, public health emergencies tend to accelerate the spread of the disease. In real life, an effective and widely used strategy to limit the spread and development of public health emergencies worldwide is to limit the large-scale flow of the population. China has controlled COVID-19 well, which is the most effective way to implement the policy and is an effective way to curb the spread of public health emergencies and to prevent the spread of the pandemic.

With the disposal method of "early detection, early isolation and early treatment" being accepted by an increasing number of countries, the means of shutdown, suspension of production, suspension of schools, road closures and even comprehensive "city closures" are carried out around the world. The implementation of an extensive segregation policy depresses the commodity consumption industry, which directly and indirectly affects the logistics and transportation industry, including dry bulk shipping.

### 3.2 Dry bulk shipping and interindustry transmission fluctuations from the perspective of the global industrial chain

**Fig 2:**
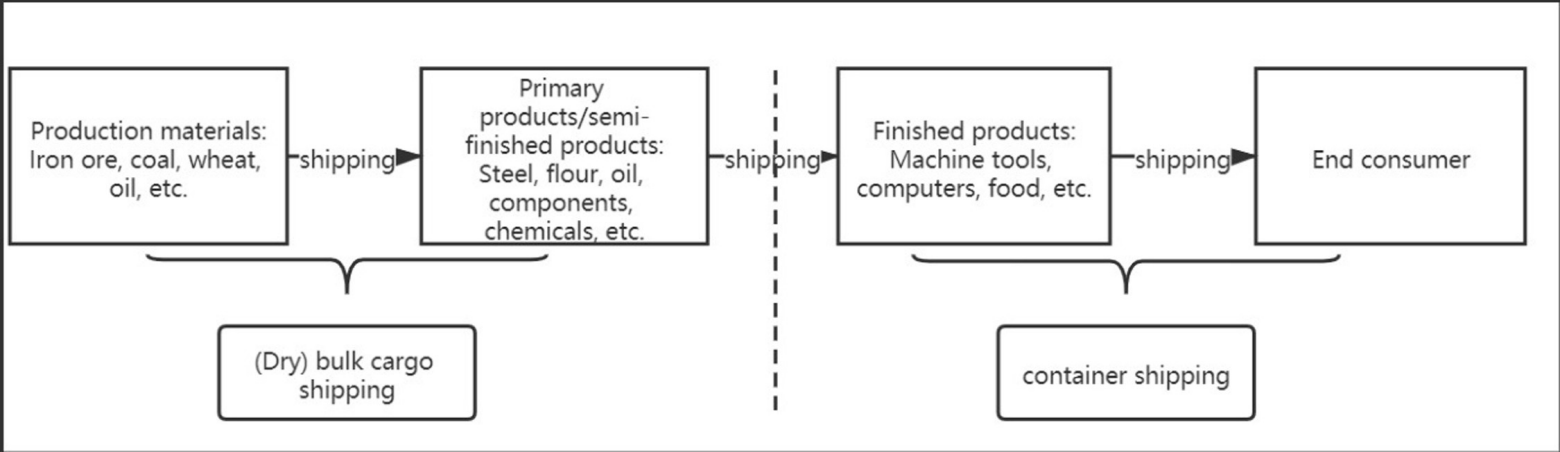
Role of dry bulk shipping in global trade.

With the further development of the internationalization strategy of the world’s large shipping enterprises, the shipping market, as a link between the means of production and the rest of the world, occupies an important position in the global supply chain. According to the statistics of the United Nations Commission for trade development, the volume of seaborne trade accounts for 90% of the total global trade in weight and for more than 70% of the trade volume in value. As shown in Fig 2, shipping is irreplaceable in global trade and regional trade. As the main transportation medium of intercontinental industrial raw materials, primary grain products and other basic primary products and semifinished products, dry bulk shipping is a key link in global economic development. There is a high correlation between dry bulk shipping and trade. Changes in trade policy, trade demand and trade framework will affect dry bulk shipping. Public health emergencies, on the one hand, obstruct the normal operation of logistics and transportation, make the communication between the production and consumer ends stagnant, and destroy the global supply chain. This will lead to a series of problems, such as shrinking consumption, creating imbalances between supply and marketing, and shrinking trade, which will seriously harm the sound development of the global economy. Furthermore, with the spread of public health emergencies, according to Maslow’s demand theory, if the population demand side cannot meet the demand for personal safety at the bottom, this is bound to cause a sharp contraction of the demand side of commodity trade. The negative feedback mechanism formed in the two aspects has greatly impacted the normal operations of dry bulk shipping.

## 4. Empirical analysis of the impact

### 4.1 Model variable processing

#### 4.1.1 Data processing

**Fig 3:**
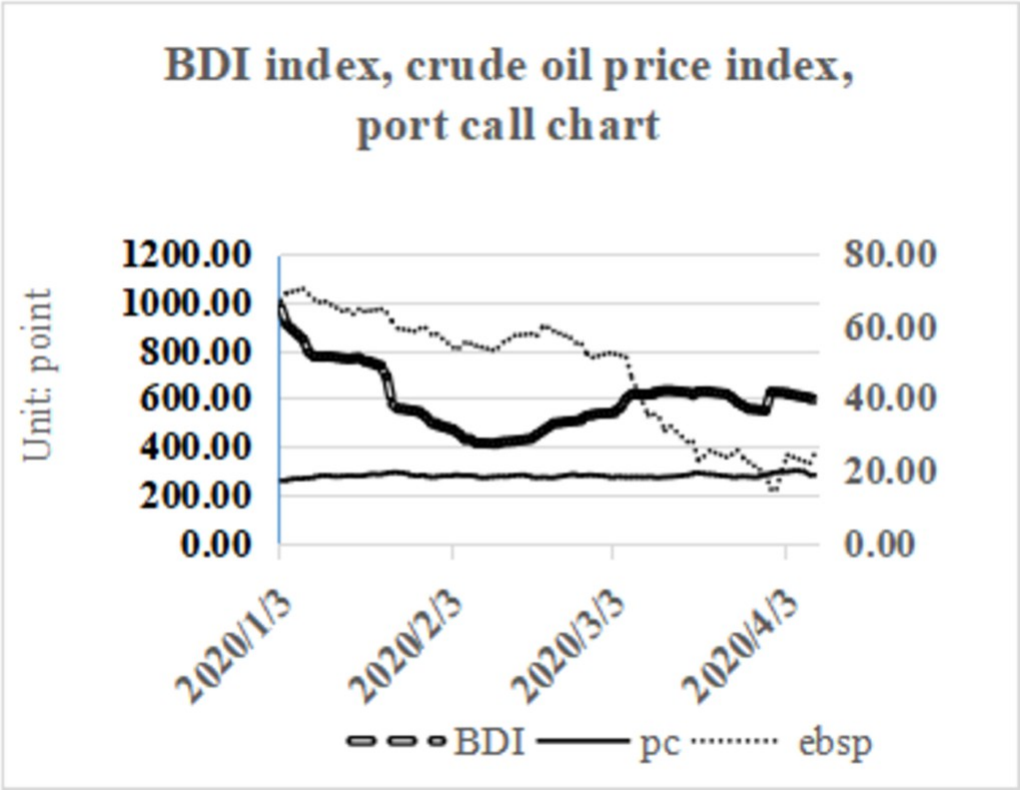
BDI index, crude oil price index, port linked trend chart.

Fig 3 shows the changes in the BDI index, European Brent crude oil price index and the number of dry bulk ships berthed at ports within the sample range. Before the COVID-19 outbreak, the BDI index was 1,380 points in December 2019. The left index system shows that the BDI index dropped rapidly to 500 after the outbreak. It can be seen from the right side of the map that the international crude oil price dropped from $67.2 per barrel in December 2019 to approximately 10 US dollars per barrel. The trends of the two indices are similar on the time axis, reflecting the linkage between the markets.

**Fig 4:**
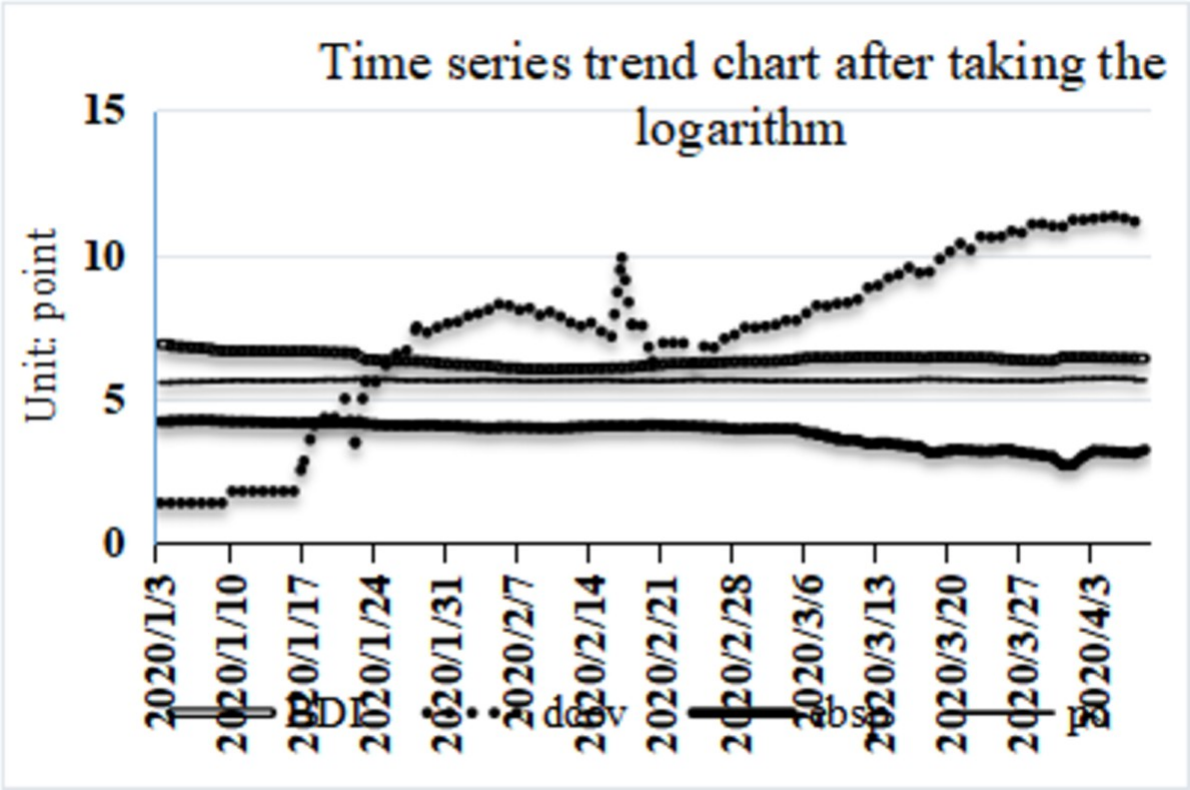
Logarithmic time series trend chart.

Due to the large volume difference between the data, to reduce the volatility of the daily data of the time series and the influence of the order of magnitude between different series, four groups of data are logarithmically processed, and the trend of the processed data is shown in Fig 4. By observing the trend chart of the processed time series data, it is considered that the logarithmic processed three series basically eliminates the influence of the order of magnitude and short-term data volatility. The COVID-19 pandemic gradually increased to a steady growth trend during the whole observation period. With the spread of COVID-19 pneumonia, the BDI index, the European Brent crude oil price index and the daily port affiliation growth rate have fluctuated to varying degrees. The BDI index representing the dry bulk shipping market shows a clear trend of "quick drop and slow down". At the same time, by comparing the crude oil market with the dry bulk shipping market, it is found that the changing trend of the crude oil market lags slightly behind that of the dry bulk shipping market. This can be explained as the later crude oil market superimposed with other shocks, including OPEC production reduction.

#### 4.1.2 Variable stationarity test

In the process of time series data analysis, because of its nonstationary characteristics, pseudoregression events often occur. To avoid pseudoregression, the stationarity of time series variables should be tested before analysis. The ADF (Augmented Dickey-Fuller test) test method is often used to test the stationarity of variables. The form of the ADF test equation is as follows

$$\Delta \left|Y_{t}\right.=\alpha +\beta t+\delta Y_{t-1}+\sum_{j=1}^{p}\lambda_{j}\Delta Y_{t-j}+\mu t$$ (1)

The results obtained by Eviews analysis software are shown in Table 1. The table shows that the t statistic values of the BDI index, the European crude oil price index and the port calls of dry bulk ships are all above the 5% confidence level threshold. The original hypothesis was accepted; that is, the series were nonstationary series. Accordingly, the t statistics of several groups of variables after the first-order difference are less than the critical value under the 1% confidence level; that is, the original hypothesis is rejected and the sequence is stable.

**Table 1:** Results of ADF test for stationarity.

	ADF test value	1% confidence level	5% confidence level	Adjoint probability p	Null hypothesis: the existence of unit root
BDI	--2.396402	-3.500669	--2.892200	0.1455	accept
COVID-19	1.643880	-3.500669	-2.892200	0.9995	accept
EBSP	-0.736219	-3.500669	-2.892200	0.8317	accept
Port Call	-2.343521	-3.500669	-2.892200	0.1609	accept
Δ BDI	-6.715796	-2.589531	-1.944248	0.0000	refuse
Δ COVID-19	-12.40858	-2.589531	-1.944248	0.0000	refuse
Δ EBSP	-6.640358	-2.589531	-1.944248	0.0000	refuse
Δ Port Call	-7.138023	-2.589531	-1.944248	0.0000	refuse

#### 4.1.3 Variable cointegration test

Using a time series to build a model, we need to test whether there is a cointegration relationship between variables based on stable data. Therefore, it is necessary to test the cointegration of the series. The cointegration test determines whether there is a long-term equilibrium relationship between time series. This paper uses the trace test method proposed by Johansen and Juselius (1990) to test the cointegration relationship between variables. The test formula is as follows (2)

$$\lambda_{trace}=-2ln\left(Q\right)-T\sum_{i=r+1}^{p}ln\left(1-{\overset{\wedge }{\lambda }}_{i}\right)$$ (2)

EViews analysis software was used to test the cointegration relationship between BDI and dcov, and the results are shown in Table 2.

**Table 2:** Cointegration relationship (Trace) test results.

Cointegration relationship	Characteristic value	Statistic	5% confidence level	Adjoint probability
r = 0	0.0813	11.417	15.494	0.1871
r≤1	0.0359	3.4460	3.8414	0.0634

Table 2 shows that there is no long-run equilibrium relationship between the 2 groups at the 5% confidence level. After the cointegration test, the BDI model and the dCOV after the first-order difference can be used as endogenous variables to construct the VAR model.

### 4.2 Model demonstration

#### 4.2.1 Establishment of VAR model

The VAR model is usually used in the prediction of time series and the dynamic impact of random disturbance on the system to explain the impact of dynamic impact on variables. The basic mathematical expression of the model is shown in Formula (3), and the meaning of the variables is shown in Table 3.


$$Y_{t}=\Gamma_{1}Y_{t-1}+\ldots +\Gamma_{p}Y_{t-p}+H_{q}X_{t}+\varepsilon_{t}$$ 3)

**Table 3:** VAR Model variable definition.

Variable	Meaning
*Y*_*t*_	Endogenous variable matrix
*X*_*t*_	Exogenous variable matrix
Γ *H*	Parameter matrix to be estimated
*ε*_*t*_	Random error term
*P*	Lag order

#### 4.2.2 Model lag order selection

The optimal lag order of the model is selected according to the AIC and SC criterion. The analysis results are shown in Table 4 by EViews measurement software.

**Table 4:** Delay order table of VAR model.

Lag	Log L	LR	FPE	AIC	SC	HQ
0	-70.59438	NA	0.019169	1.721222	1.888995	1.788847
1	127.39	373.7233	0.000245	-2.637978	-2.358356	-2.52527
2	143.2823	29.28469*	0.000188*	-2.905220	-2.513750*	-2.747430*
3	144.5467	2.273094	0.0002	-2.843747	-2.340427	-2.640873
4	145.8485	2.281793	0.000213	-2.783113	-2.167944	-2.535156
5	147.2901	2.461962	0.000226	-2.725619	-1.998602	-2.432579
6	149.151	3.094615	0.000237	-2.677551	-1.838685	-2.339428
7	151.7372	4.184334	0.000246	-2.645779	-1.69506	-2.26257
8	152.614	1.379226	0.000264	-2.57559	-1.513031	-2.147305

Through the analysis of Table 4, it is found that the optimal lag order under the AIC is on the order of 2, and the optimal lag order under the SC criterion is on the order of 2. Combining the LR test, FPE test and HQ test, it is determined that the optimal lag order of the model selected in this paper is order 2.

The optimal model Eqs (4) and (5) are obtained as follows:

$$\begin{array}{l} \text{ln}\;BDI=1.29*\text{ln}\;BDI\left(-1\right)-0.39*\text{ln}\;BDI\left(-2\right)+0.01*\text{ln}\;COV\left(-1\right)\\ -0.02*\text{ln}\;COV\left(-2\right)-0.06*\text{ln}\;EBSP-0.18*\text{ln}\;PC+1.97 \end{array}$$ 4)

$$\begin{array}{l} \text{ln}\;COV=-2.41*\text{ln}\;BDI\left(-1\right)+2.01*\text{ln}\;BDI\left(-2\right)+0.50*\text{ln}\;COV\left(-1\right)\\ +0.40*\text{ln}\;COV\left(-2\right)-0.49*\text{ln}\;EBSP+2.20*\text{ln}\;PC-7.11 \end{array}$$ 5)

#### 4.2.3 Granger causality test

The Granger causality test is often used to judge the causality between variables, that is, whether the change of one variable is the cause of the change of another variable. On this basis, the causal direction of the two is tested. Next, the optimal VAR model calculated above is used for the causality test. The inspection results are shown in Table 5.

**Table 5:** Granger causality test results.

Original hypothesis	F statistic	P value
lnCOV is not the Granger cause of lnBDI	10.88	0.0043
lnBDI is not the Granger cause of lnCOV	1.74	0.4199

According to Table 5, the probability of the COVID-19 pandemic explaining the fluctuations in the dry bulk shipping market is 0.9957, while the probability of dry bulk shipping market fluctuations explaining the development of the COVID-19 pandemic is only 0.5801, which is not significant. In other words, there is a unidirectional causal relationship between the two markets.

Granger causality test results are consistent with the development of reality and corroborate the impact of the COVID-19 public health emergency on the dry bulk shipping market. The global spread of public health emergencies has a direct impact on the trade side. At the same time, due to the strong demand and high dependence on seafarers and other means of production, the dry bulk shipping market has had a relatively strong impact. The COVID-19 pandemic was mainly affected by the policies of various countries and regions in the sample period and was closely related to passenger transport. The development of a dry bulk shipping market dominated by cargo transport is not significant. Under the background of global supply chain development, the dry bulk shipping market is strongly correlated with the development of public health emergencies.

#### 4.2.4 SVAR model estimation and recognition

In the analysis of the VAR model, the real-time structural relationship among system variables is hidden in the random disturbance term, and the explanation of this part is ignored. The SVAR model adds synchronous variables to the VAR model, which can directly reflect the real-time structural relationship among variables [26]. The BDI index and other variables reflecting market price changes will quickly absorb the impact of current related variables and give feedback on the changes of current related variables. Considering the characteristics of this kind of data, the analysis depth of the VAR model established only by considering the lag effect of endogenous variables is insufficient. The basic expression of the SVAR model is shown in Formula (6)

$$Y_{t}={\overset{\wedge }{\Gamma }}_{1}Y_{t-1}+\cdots +{\overset{\wedge }{\Gamma }}_{p}Y_{t-p}+{\overset{\wedge }{H}}_{q}X_{t}+\mu_{t}$$ (6)

Among them, matrix A is the synchronous relation matrix between endogenous variables in the model, and it is the white noise vector.

Formula (7) is obtained by multiplying both sides of the VAR model by the synchronous relation matrix A at the same time.


$$AY_{t}=A\Gamma_{1}Y_{t-1}+\cdots +A\Gamma_{p}Y_{t-p}+AH_{q}X_{t}+A\varepsilon_{t}$$ (7)

Formula (8) can be obtained by simultaneous Formulas (6) and (7)

$$A\varepsilon_{t}=\mu_{t}$$ (8)

After orthogonalization, that is, orthogonalizing *μ*_*t*_ to *μ*_*t*_ = *Be*_*t*_, the estimation model Formula (9) of the AB-type SVAR model is obtained:

$$A\varepsilon_{t}=Be_{t}$$ (9)

In this paper, the SVAR model is identified by establishing short-term constraints. Because the model includes two endogenous variables, it needs to impose *m*(*m*−1)/2 constraints, namely, one constraint. According to the economic meaning, the restriction condition is as follows: The development of COVID-19 has a current effect on the freight rate of the dry bulk shipping market.

After setting constraints and sorting out, the form of Eq (9) is matrix Eq (10):

$$\left(\begin{array}{cc} 1 & 0\\ a_{21} & 1 \end{array}\right)\left(\begin{array}{c} \varepsilon_{lnBDI}\\ \varepsilon_{lnCOV} \end{array}\right)=\left(\begin{array}{cc} b_{11} & 0\\ 0 & b_{22} \end{array}\right)\left(\begin{array}{c} e_{lnBDI}\\ e_{lnCOV} \end{array}\right)$$ (10)

After importing EViews measurement software, the estimated values of matrix parameters and related data are obtained after maximum likelihood estimation, as shown in Table 6. The estimated values of the model are significant at the 5% confidence level, and the model fitting effect is good.

**Table 6:** SVAR model parameter estimate.

Matrix parameters	Estimated value	Standard deviation	Z statistic	Probability
A_21_	4.041	1.914	2.110	0.035
B_11_	0.026	0.002	13.781	0.000
B_22_	0.483	0.035	13.781	0.000

#### 4.2.5 Impulse response analysis among variables

**Fig 5:**
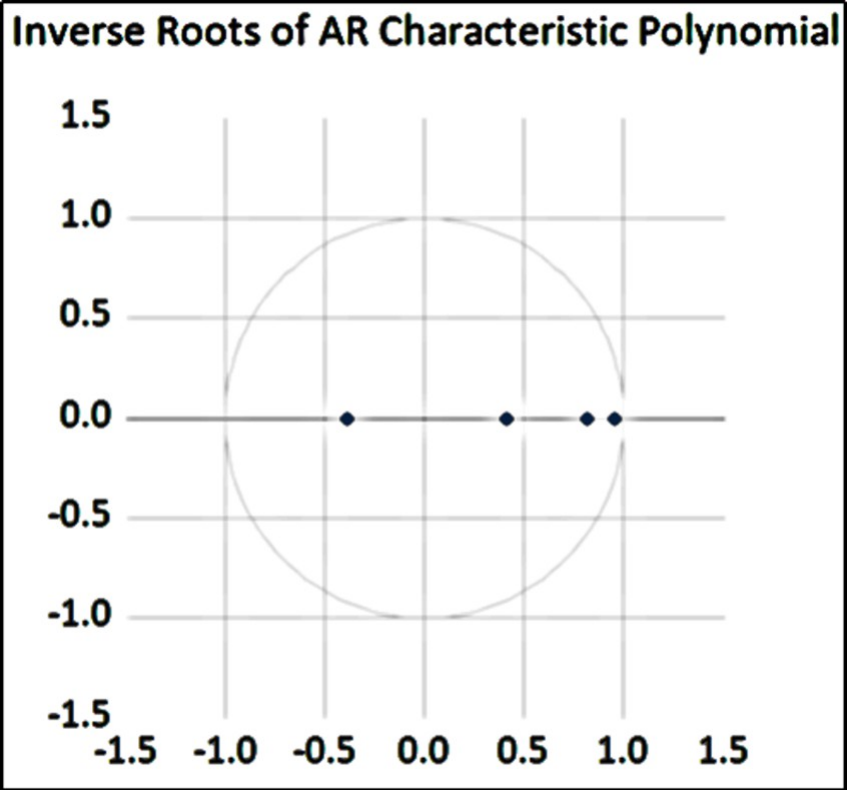
AR root test diagram.

By exploring the impulse response of the SVAR model, we can investigate the impact effect of public health emergencies on dry bulk shipping in different periods. The premise of the impulse response is that the model is stable, so it is necessary to carry out AR root tests on the model to judge whether the model is stable or not. The AR root test results are shown in Fig 5. The characteristic roots of the VAR model are located in the unit circle, and the reciprocal values of the characteristic roots are less than 1, so the model is stable.

Based on the stability of the model, the impulse response is analyzed. The purpose of this study is to analyze the dynamic impact of the COVID-19 public health emergency on the dry bulk shipping market represented by the BDI index. The solid line in Fig 5 is the impulse response trend shown by the impulse response function.

**Fig 6:**
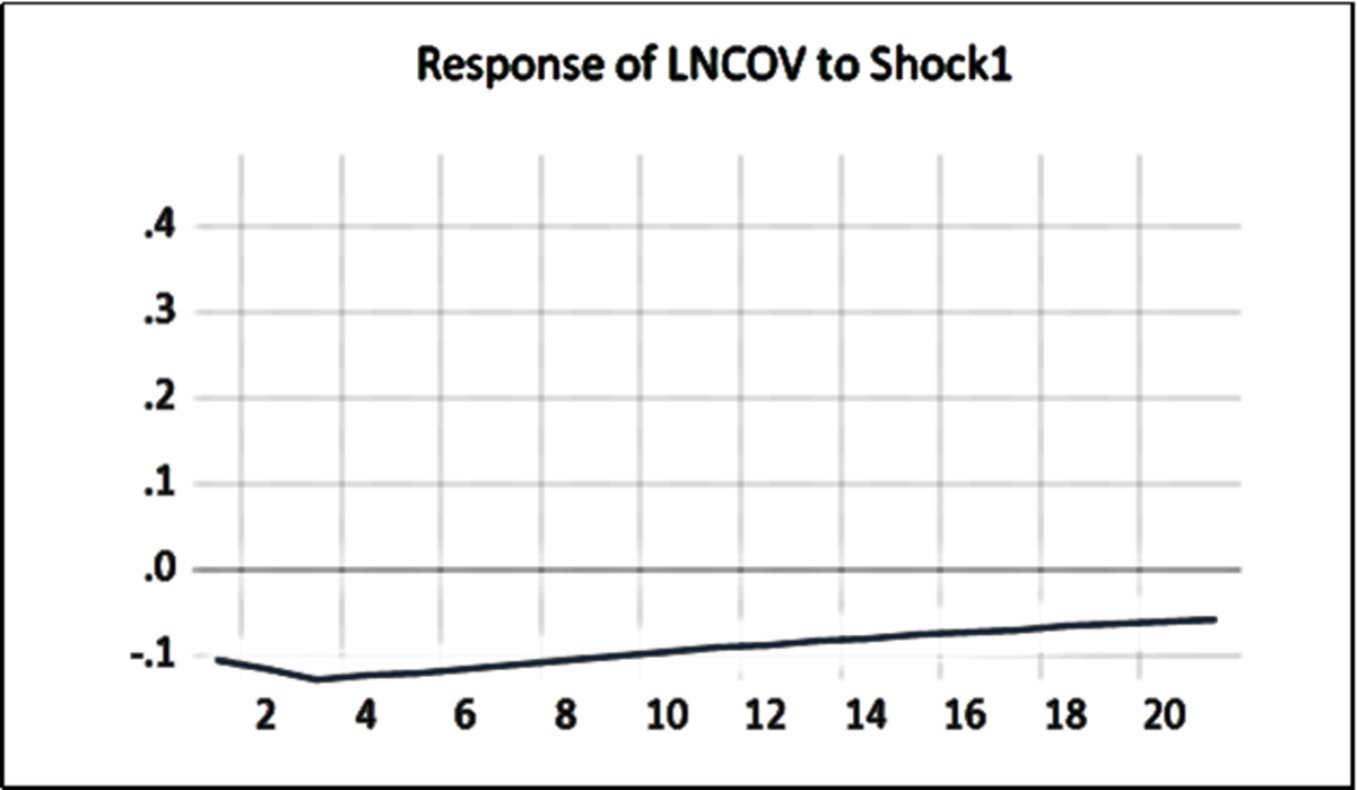
Model impulse response diagram.

The COVID-19 pandemic has a significant impact on the dry bulk shipping market; the impact duration is more than 3 weeks, and it always has a negative impact, as shown in Fig 6. The impact increases gradually in the early stage, reaches the maximum strength in the third stage, and then decreases gradually but does not disappear, and there is a long-term lasting impact. The impact of the increase of confirmed cases of COVID-19 on the dry bulk shipping market and related enterprises reached the highest point in a short period (1–3 days), and then the impact of this period will gradually decrease, but it will continue for more than 3 weeks. COVID-19 will continue to increase the cost of dry bulk shipping, and the demand for dry bulk shipping will decrease, making it difficult for dry bulk shipping enterprises to continue. The aggravation of the pandemic seriously affected the market, which was finally reflected by the weakness of the BDI index. The dynamic impact reached the maximum negative impact in the third period (the third day) and then showed a downward trend, affecting dry bulk shipping for a long time.

#### 4.2.6 Model variance decomposition analysis

As shown in the variance decomposition chart of Table 7, the impact contribution rate of the BDI index itself is approximately 62%, and the impact contribution rate of the pandemic and other factors is approximately 38%, which shows that the dry bulk shipping market and related shipping enterprises are highly affected by external factors. Dry bulk shipping was deeply impacted by the COVID-19 public health emergency.

**Table 7:** Variance decomposition table.

Variance decomposition of lnBDI			
	S.E.	lnBDI (Shock1)	lnCOV (Shock2)
1	0.0259	100.00%	0%
2	0.0419	99.49%	0.51%
3	0.0533	99.54%	0.46%
4	0.0618	98.84%	1.16%
5	0.0684	97.14%	2.86%
6	0.0739	94.74%	5.26%
7	0.0787	91.83%	8.17%
8	0.0829	88.70%	11.30%
9	0.0868	85.51%	14.49%
10	0.0903	82.40%	17.60%
11	0.0935	79.47%	20.53%
12	0.0965	76.75%	23.25%
13	0.0993	74.26%	25.74%
14	0.1018	72.00%	28.00%
15	0.1042	69.97%	30.03%
16	0.1064	68.14%	31.86%
17	0.1083	66.51%	33.49%
18	0.1102	65.04%	34.96%
19	0.1118	63.74%	36.26%
20	0.1133	62.57%	37.43%
21	0.1147	61.52%	38.48%

## 4.3 Conclusion of empirical analysis

By studying the impact of the COVID-19 pandemic’s public health emergency on the BDI index, we discovered that there was no cointegration relationship between the BDI index and newly confirmed pneumonia cases related to COVID-19 at the 5% confidence level. The Granger causality test showed a one-way causal relationship between the COVID-19 pandemic and the dry bulk shipping market. Impulse response analysis showed that dry bulk shipping was significantly negatively impacted by the volatility of confirmed new cases of COVID-19, with a duration of more than 3 weeks. The increase in the impact of COVID-19 confirmed cases’ reached the highest point in the short term (1–3 days) for the dry bulk shipping market and related enterprises. The impact will gradually decrease, but it will last more than 3 weeks. The result of variance decomposition proved that dry bulk shipping was deeply impacted by the COVID-19 public health emergency.

Thus, the dry bulk shipping market quickly received external shocks by the COVID-19 pandemic. Dry bulk shipping enterprises need to be alert that the external impact will reach the maximum in the short term (1–3 days) and will last more than 3 weeks in the long term. Before taking effective measures, the impact of public health emergencies will continue to impact the market in waves, greatly affecting the performance and cash flow of enterprises.

## 5. Analysis of risk transfer framework

### 5.1 Spillover index and incidence matrix

Diebold and Yilmaz (2014) proposed the spillover index method based on generalized prediction error variance decomposition to measure the risk spillover effect among financial sectors and then described the risk transmission relationship among sectors [27]. The model construction process is as follows: Based on the constructed n-dimensional p-order VAR model, generalized variance decomposition is carried out, and the total spillover index and directional spillover index are constructed by using the variance decomposition results after calculation and processing [28]. Finally, the network topology method proposed by Diebold and Yilmaz is used to construct the incidence matrix.

Taking the logarithm of the return index of each dry bulk shipping market segment, the logarithmic rate of return of each ship type market segment is obtained, generalized variance decomposition is carried out, and the risk volatility spillover index is obtained after calculation. On this basis, the risk volatility spillover correlation matrix of the dry bulk shipping subdivision ship type is constructed.

In Table 8, the matrix row and column elements are explained as the risk volatility spillover effect strength of each column variable to the row variable, and the total risk volatility spillover strength is shown in the lower right corner. All values in the “from” column represent the sum of the risk volatility spillover effects of the corresponding row variables from all market segments except itself. The “to” line is the sum of the risk volatility spillover effects of the corresponding variables on all other market segments except itself. The net volatility spillover index row represents the net risk volatility spillover index of the corresponding column variables. A positive or negative value indicates that the segment is a net exporter or importer of risk volatility, respectively.

**Table 8:** Matrix representation of the degree of association.

Incidence matrix	x_1_	x_2_	…	x_n_	From
x_1_	$d_{11}^{H}$	$d_{12}^{H}$	…	$d_{1n}^{H}$	$\sum_{j=1}^{n}d_{1j}^{H},j\neq 1$
x_2_	$d_{21}^{H}$	$d_{22}^{H}$	…	$d_{2n}^{H}$	$\sum_{j=1}^{n}d_{2j}^{H},j\neq 2$
…	…	…	…	…	…
x_n_	$d_{n1}^{H}$	$d_{n2}^{H}$	…	$d_{nn}^{H}$	$\sum_{j=1}^{n}d_{nj}^{H},j\neq n$
To	$\sum_{i=1}^{n}d_{i1}^{H},i\neq 1$	$\sum_{i=1}^{n}d_{i2}^{H},i\neq 2$	…	$\sum_{i=1}^{n}d_{in}^{H},i\neq n$	Total Risk Spillover intensity: * * *%
volatility spillover index NET	$\sum_{i=1}^{n}d_{i1}^{H}-\sum_{j=1}^{n}d_{1j}^{H},ij\neq 1$	$\sum_{i=1}^{n}d_{i2}^{H}-\sum_{j=1}^{n}d_{2j}^{H},ij\neq 2$	…	$\sum_{i=1}^{n}d_{in}^{H}-\sum_{j=1}^{n}d_{nj}^{H},ij\neq n$	

### 5.2 Analysis of the risk fluctuation transfer framework

#### 5.2.1 Analysis of risk fluctuation in the study period of the COVID-19 pandemic

By comparing the performance of the shipping rate index in the years before and after the public health emergency, the impact of the COVID-19 pandemic on the dry bulk shipping market is examined more directly to lay a structural foundation for an in-depth analysis of risk transfer between market segments. The outbreak of the COVID-19 pandemic is a worldwide public health event that affects worldwide demand for major countries in international trade and major sources of production. The impact on dry bulk shipping is reflected not only in the contraction of transport prices but also in the transport structure of dry bulk shipping. As shown in Table 9, by comparing the performance of the freight rate in 2019 and 2020 before and after the outbreak of the COVID-19 pandemic, the index of Handysize, Panamax and capsize dry bulk carriers fell by 16.92%, 24.56% and 38.94%, respectively. Under the overall pressure of the dry bulk shipping market, capsize dry bulk carriers are the first to bear the brunt. In the first three quarters of 2020, the average freight rate of capsize dry bulk carriers decreased by 38.94% monthly, and in the first quarter of 2020, the freight rate of cape dry bulk carriers decreased by 90.34% monthly. After the outbreak of COVID-19, the transport structure of the dry bulk shipping market was greatly affected, and the volume of trade decreased significantly, which directly impacted the volume of dry bulk cargo. The revenue of the capsize dry bulk carrier, which should represent a significant proportion in the market, was negative for an extended period in the first quarter.

**Table 9:** Comparison of market performance of different ship types before and after the COVID-19 pandemic.

quarter	BHI			BPI			BCI		
	2019	2020	Volatility	2019	2020	Volatility	2019	2020	Volatility
First quarter	413.8	361.9	-12.54%	871.6	786.7	-9.74%	964.4	93.2	-90.34%
Second quarter	406.8	290	-28.71%	1188.8	821.1	-30.93%	1309.7	1132.6	-13.52%
Third quarter	575	507.5	-11.74%	2001.6	1456.4	-27.24%	3870.8	2526.2	-34.74%
Average value	465.2	386.5	-16.92%	1354	1021.4	-24.56%	2048.3	1250.7	-38.94%

Using spillover index analysis, we obtained the risk volatility spillover matrix of the dry bulk shipping market during the period of COVID-19. Table 10 shows that the capsize shipping market and handy size shipping market are net risk acceptors, and the Panamax shipping market is a net risk spillover, according to the risk spillover rate ranking—Panamax type, super handy size type, and capsize type—and according to the risk acceptance rate ranking—handy size type, capsize type, and Panamax type. The volatility index of the risk spillover of the dry bulk shipping market is 20.1%. The 2019 COVID-19 pandemic outbreaks overlapped with the Spring Festival holidays in China. Large ships such as the capsize dry bulk shipping market are more sensitive to cargo volume due to their high volume and low marginal cost. At that time, the capsize dry bulk shipping market index rapidly dropped from more than 1,500 points to a negative value in January 2020, and the market carrying ratio dropped rapidly, which was reflected in the data as the net risk importer. With the deepening of resumption of production worldwide, Panamax dry bulk carriers have undertaken a considerable part of the carrying function of capsize. The Panamax dry bulk shipping price index entered the recovery period relatively early, which also proves this point.

**Table 10:** Risk volatility spillover table of the dry bulk shipping market during the study period of COVID-19.

COVID-19	(Super) handy size dry bulk shipping market	Panamax dry bulk shipping market	Capsize dry bulk shipping market	From
(Super) handy size dry bulk shipping market	62.8	31.4	5.7	37.2
Panamax dry bulk shipping market	1.7	97.1	1.2	**2.9**
Capsize dry bulk shipping market	8.3	11.8	79.9	20.1
To	10.1	**43.2**	6.9	Total Risk Spillover intensity: 20.1%
Net volatility spillover index	-27.1	**40.3**	-13.2	
Net risk input/output	Net input	Net output	Net input	

#### 5.2.2 Analysis of risk fluctuation in the study period of the Zika virus epidemic

The incidence of Zika virus infection is relatively small compared to that of COVID-19, and it mainly affects the place of origin of international trade production, which is not the only one. By observing the changes in the freight index of dry bulk shipping in the first three quarters of 2015 and 2016 before and after the outbreak of the Zika virus, it can be seen in Table 11 that during the study period of the Zika virus epidemic, the freight index of dry bulk shipping was impacted somewhat, with freight fluctuation decreasing. The freight rates of the three major ship types fell by 15.32%, 29.24% and 24.36%. The impact of the Zika virus epidemic is different from that of COVID-19, and the impact of several ship type markets is relatively close. The impact of public health emergencies is mainly reflected in the pressure on overall transport volume and freight rates, but the impact on the overall transport structure is not obvious.

**Table 11:** Comparison of market performance of different ship types before and after the Zika virus epidemic.

	BHI			BPI			BCI		
	2015	2016	Volatility	2015	2016	Volatility	2015	2016	Volatility
First quarter	358.8	230.3	-35.81%	602.4	382.9	-36.44%	556.5	221.t	-60.14%
Second quarter	345.8	332.6	-3.82%	649.6	613.4	-5.57%	786.5	882.9	12.26%
Third quarter	431.5	399.2	-7.49%	954.3	565	-40.79%	1682.8	1184.1	-29.64%
Average	378.7	320.7	-15.32%	735.4	520.4	-29.24%	1008.6	762.9	-24.36%

By using spillover index analysis, the risk volatility spillover matrix of each ship type in the dry bulk shipping market during the Zika epidemic is obtained, as shown in Table 12. It can be seen that during the Zika epidemic, the capsize shipping market is the net risk spillover side, and the (super) handy size and Panamax shipping market are the net risk acceptor side, according to the risk spillover rate ranking—capsize type, Panamax type, and (super) handy size type—and according to the risk acceptance rate ranking—Panamax type, (super) handy size type, and capsize type. The overall market risk spillover volatility index of the dry bulk shipping market is 11.8%. As the Zika epidemic did not have a great impact on the leading demand countries of global trade dry bulk shipping or the main supply countries except Brazil, the public health emergency did not have a great impact on the overall development of world trade. At the same time, the Zika epidemic only affected Brazil, which is not the only producer of basic means of production. It is superimposed with strong trade demand under the development of the world economy. Each market segment of dry bulk shipping only affects traffic volume, not the transport structure.

**Table 12:** Risk volatility spillover table of the dry bulk shipping market during the study period of the Zika epidemic.

Zika	(Super) handy size dry bulk shipping market	Panamax dry bulk shipping market	capsize dry bulk shipping market	From
(Super) handy size dry bulk shipping market	89.5	7.6	2.9	10.5
Panamax dry bulk shipping market	3.4	82.9	13.7	17.1
Capsize dry bulk shipping market	3.3	4.5	92.2	**7.8**
To	6.7	12.1	**16.6**	Total Risk Spillover intensity: 11.8%
Net volatility Spillover index	-3.8	-5	**8.8**	
Net risk input/output	Net input	Net input	Net output	

#### 5.2.3 Comparative analysis

**Fig 7:**
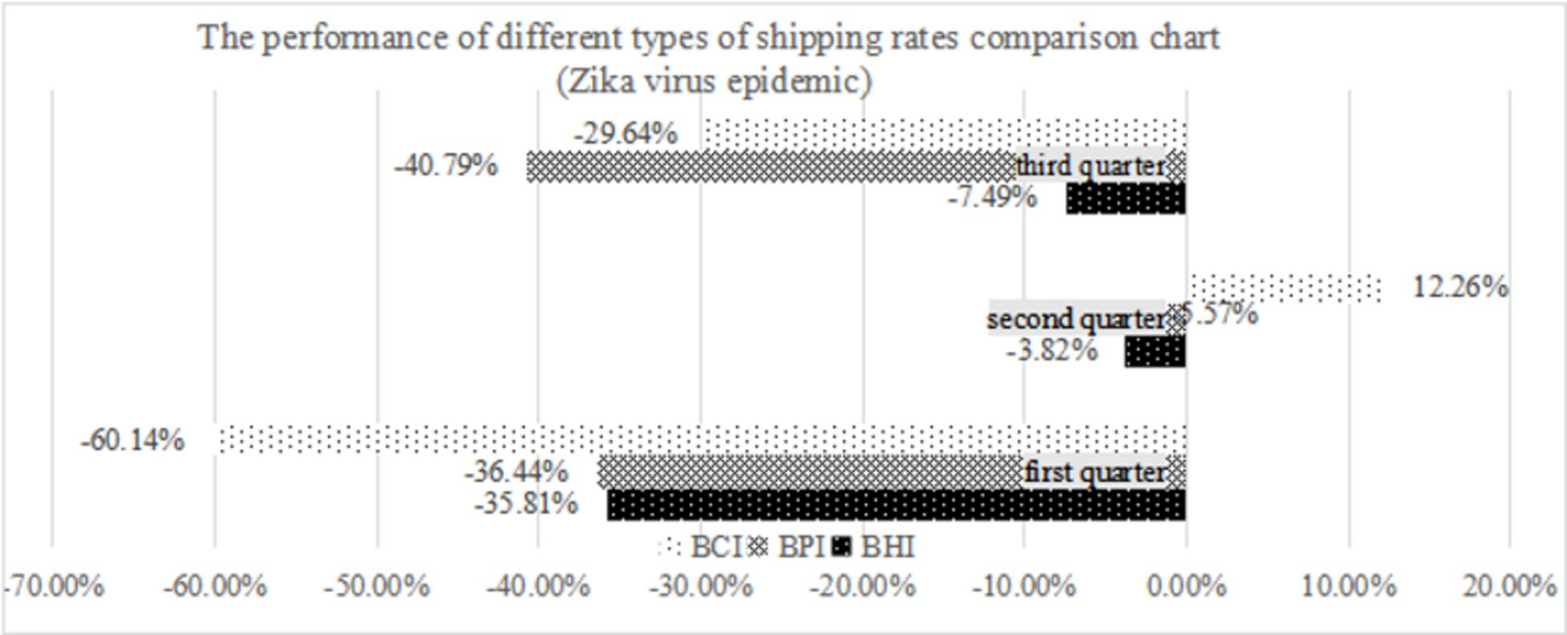
The performance of different types of shipping rate comparison charts (Zika virus epidemic).

**Fig 8:**
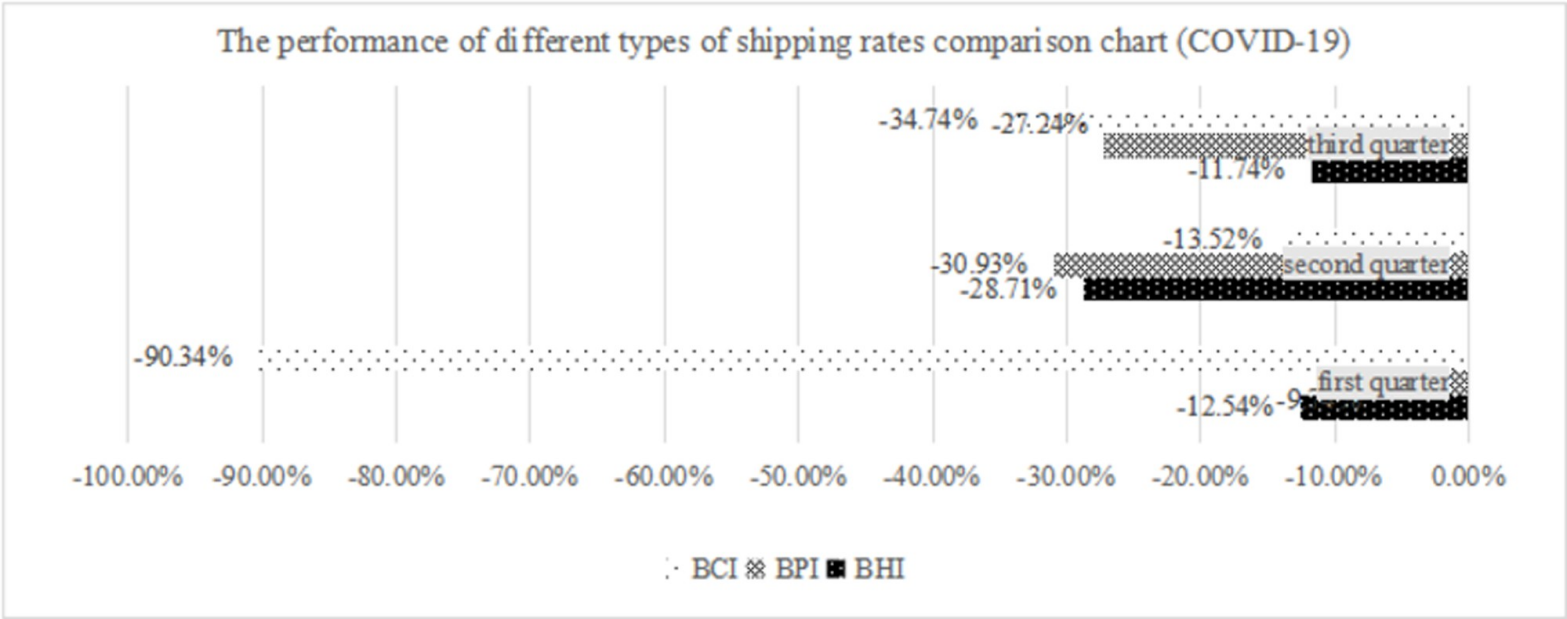
The performance of different types of shipping rate comparison charts (COVID-19).

Comparing the freight rate performance of each shipping type before and after the outbreak of two public health emergencies and comparing Figs 7 and 8, we can draw the conclusion that public health emergencies have indeed caused different degrees of impact on the dry bulk shipping market. Large ships such as capsize dry bulk carriers are more vulnerable when the volume of bulk cargo in international trade fluctuates. When the scope of public health emergencies is large and extends to both sides of international trade supply and demand, the impact is more obvious. The capsize-type shipping freight rate fell by approximately 60% in the first quarter of the Zika virus epidemic, and the freight rate fell by approximately 90% in the first quarter of the COVID-19 pandemic. At the same time, serious public health emergencies may affect the transport structure of the dry bulk shipping market.

Considering the overall risk volatility spillover of the dry bulk shipping market, by comparing the risk volatility spillover in Tables 10 and 12 with the development of the world economy, the market connection between the segmented shipping type markets in the dry bulk shipping market is closer, and the volatility index of risk spillover between markets increased significantly, from 11.8% to 20.1%. This shows that with the development of shipping finance and other industries, the relationship between different segments of the dry bulk shipping market have become more closely connected.

During the Zika outbreak in 2016, the increase of iron ore shares on China–Brazil routes helped increase demand, the further development of intermediate frequency furnace events positively impacted the import demand of iron ore, and the large-scale steel-making movement in India increased the demand for coking coal carbon trade, in response to which the demand for dry bulk shipping rose steadily. At a time when the large-scale development of ships is in full swing, capsize dry bulk shipping has made great progress, and the market enjoys the benefit of marginal cost reductions. With the advantages of high deadweight tonnage, strong adaptability, and low unit cost, the capsize dry bulk carrier occupies a dominant position in the dry bulk shipping market. According to the market rule of large ships with large lines, the market position of Panamax dry bulk carriers with relatively large deadweight tonnage is closely followed by (Super) handy size dry bulk shipping. At that time, the internal risk to the dry bulk shipping market was mainly due to the transfer of the capsize dry bulk shipping market segment to the Panamax type and (super) handy size type dry bulk shipping market segments. The Zika virus epidemic, as a typical public health emergency in noncore supply and demand countries of world trade, has a relatively low impact on world trade. As reflected in dry bulk shipping, it mainly acts on the supply side of both sides of trade demand and has a relatively limited impact on Brazil’s iron ore trade.

During the period of analysis of the COVID-19 pandemic, the BCI index was negative for the first time in history; that is, capsize dry bulk shipping was not profitable during that period. In addition to the seasonal impact, the main reason is the sharp reduction of international demand. China is one of the major demand countries for ’s iron ore trade, the demand for which dwindled considerably during COVID-19. Accordingly, Brazil, as one of the main suppliers of iron ore trade, was affected by the pandemic, and the delivery recovery was slow. Compared with the same period, Brazil’s grain exports also decreased by 11% in January and February 2020. The capsize shipping market is still in the doldrums, but market demand has been maintained. The smaller Panamax, Super Handysize and Handysize ships recovered relatively more quickly from the loss of freight rates. According to the data, the Panamax dry bulk shipping market segment, as the net spillover part of risk fluctuation, transfers risk to the capsize and (super)handy size dry bulk shipping market segment fluctuation during the period of analysis. The COVID-19 global public health emergency not only acts on the main suppliers of international trade but also affects the principal demand sides, impacting both the supply and demand of international trade, the dry bulk shipping market and related enterprises.

Considering the risk volatility spillover between two segments of the dry bulk shipping market, Table 13 shows risk volatility spillover according to the risk volatility spillover strength ranking between two segments. The risk spillover intensity table shows that the strongest risk spillover is from the Panamax dry bulk shipping market to the (Super) Handysize dry bulk shipping market during the COVID-19 pandemic. The risk spillover index is 31.4, which is generally higher than that of other markets. This can be explained by the fact that COVID-19 is a worldwide public health emergency, which is different from the Zika virus epidemic. It has a great impact on the dry bulk shipping transport structure. Due to the high overlap of dry bulk cargo transportation, there is a strong linkage between Panamax and (Super) Handysize dry bulk shipping in undertaking Capsize dry bulk shipping transportation. As the highest market heat that carried a relatively large volume of cargo, Panamax dry bulk carriers were the most important risk exporters during the COVID-19 pandemic. The weakest spillover effect is from capsize to the Panamax dry bulk shipping market, and the spillover intensity is only 1.2

**Table 13:** Risk spillover intensity of two markets during public health emergencies.

COVID-19 study period				Zika study period			
order	Risk Exporter	Risk Acceptor	Risk Spillover strength	order	Risk Exporter	Risk Acceptor	Risk Spillover strength
**1**	**Panamax**	**(super)handy size**	**31.4**	**1**	**capsize**	**Panamax**	**13.7**
**2**	Panamax	capsize	11.8	**2**	Panamax	(super)hand size	7.6
**3**	(super)Hand size	capsize	8.3	**3**	Panamax	capsize	4.5
**4**	Capsize	(super)hand size	5.7	**4**	(super)hand size	Panamax	3.4
**5**	(super)Hand size	Panamax	1.7	**5**	(super)hand size	capsize	3.3
**6**	Capsize	Panamax	1.2	**6**	capsize	(super)hand size	2.9

During the Zika epidemic, the transportation structure of dry bulk cargo did not change. The strength and direction of risk transmission are highly consistent with the volume of goods. Table 13 shows that the main risk volatility spillover chain reactions, Panamax and (Super) Handysize, 13.7 and 7.6, respectively. The weakest spillover effect is from capsize to (Super) Handysize dry bulk shipping market, and the spillover intensity is only 2.9

### 5.3 Analysis and conclusion

Through the analysis of the risk transfer framework of two typical public health emergencies, we can draw the following conclusions:

1. With the development of globalization, the relationship between the ship type market segments of the dry bulk shipping market is closer, and the degree of risk spillover is higher.
2. When public health emergencies affect only individual nodes of global trade, especially the nonunique supply side, the impact of public health emergencies on the dry bulk shipping market will not affect its transportation structure. The main risk volatility spillover chain reaction is: capsize, Panamax and (Super) Handysize.
3. When public health emergencies affect the whole process of global trade, especially regarding nodes with high demand, the dry bulk shipping market will affect the transport structure under the impact of public health emergencies. The market of the Capsize dry bulk carrier with high carrying capacity and low marginal costs will be greatly affected, and its cargo-carrying function will be replaced by a relatively more efficient small carrier with low cargo demand in the short term. In the beginning, the risk transferred rapidly to the Capsize shipping market and then mainly exports from the Panamax ship market to the (Super) Handysize ship market and the Capsize shipping market.

## 6 Conclusions

In this paper, we find that the supply and demand of the dry bulk shipping market are impacted by the COVID-19 public health emergency. Public health emergencies have a strong impact on dry bulk shipping directly or indirectly from the perspective of policy constraints and the global industrial supply chain. The research shows that the impact of a certain period will reach the maximum on the third day, after which it will gradually decrease as the pandemic comes under control, and the impact will last for more than three weeks.

Different types of public health emergencies have different impacts on the dry bulk shipping market due to their different areas of influence. The economic status of capsize dry bulk carriers will be affected by the "big event”, and the efficiency of smaller Panamax dry bulk carriers will be improved. Therefore, dry bulk shipping enterprises should accurately grasp the development direction of public health emergencies, accurately study and judge the types of events, reasonably carry out business and organize distribution and ship allocation.

The pandemic will not disappear. With the further development of the pandemic, the future perspective will focus more on exploring the opportunities and challenges of the global shipping industry and trade structure in the later stage of public health emergencies.

For dry bulk shipping companies, the most important thing is to make a quick judgment when public health emergencies occur, determine the speed of the spread and control the situation. First, we fully respond to the regional and knock-on effects of public health emergencies, summarize, and analyze the impact of world trade and customs clearance efficiency to judge whether it will impact the transportation structure of the dry bulk shipping market.

While making a good market estimation, it is important to adjust the business according to whether it will affect the dry bulk shipping structure. When the impact of public health emergencies is small and controllable or occurs in nonunitary supply markets and secondary demand markets (hereinafter referred to as "minor events"), dry bulk shipping enterprises should strengthen the management of corresponding regional routes. Flexibly adjusting transport capacity and reducing the loss of capital and income also reduces the risk of becoming the medium of transmission. When public health emergencies are strong and uncontrollable or occur only in producer countries of the means of production and the main demand side of trade (hereinafter referred to as "major events"), dry bulk shipping enterprises should quickly integrate market information, analyze the future market volume of bulk commodities and other information, and design a good distribution plan in advance. At the same time, plans should be made for possible crew shortages and becoming isolated islands at sea to eliminate having to passively accept the situation when public health emergencies occur.

In response to external risk input at the same time, dry bulk shipping enterprises should control internal risk transfer, avoid a strong impact on enterprises’ internal business between the development of the implicated role and affecting business revenues of the whole enterprise. According to the above analysis, when faced with "small events", risk transfer generally follows the principle that volume determines risk. In this situation, we should do a good job of splitting the internal business, controlling cash flow, reducing cash flow losses of damaged business, and minimizing losses. When facing "big events", enterprises should be prepared for the sudden reduction of cargo volume and the transformation of economic ship type. In the business planning of route ship allocation and ship cabin allocation, we should carry out the work of small ship types to large ship types, try to balance profits and losses by minimizing the reduction of the small ship market and help enterprises tide over the difficulties.

In addition, from the perspective of enterprises, we can also make full use of the hedging forecasting tools of financial markets. By means of financial risk prediction, we can enhance the sensitivity of enterprise risk prediction and avoid risks in shipping financial markets in advance.

### Policy implications

1. The government should strengthen plans, ensure the transparency of public health emergency information to the greatest extent possible, help the market establish and improve emergency early warning mechanisms and shorten response times.
2. The government should refine financial markets while reasonably controlling speculation, increase support for risk-averse financial products, establish and improve "rescue mechanisms", and help different subjects in sudden public health crises reduce the risk of capital chain ruptures.
3. On the premise of respecting the main role of the market, the government should strengthen guidance, develop warning and guiding standards for the rational allocation of global shipping capacity, and strive to create a dynamic and reasonable capacity pool stock.
4. The government should improve shipping laws and regulations, improve the good operation of shipping enterprises to safeguard their legitimate rights and interests and deal with the aftermath of bankruptcy according to the law and the stable mechanisms of market access, and effectively carry out standardization and supervision.
